# Immunoassays Based on Hot Electron-Induced Electrochemiluminescence at Disposable Cell Chips with Printed Electrodes

**DOI:** 10.3390/s19122751

**Published:** 2019-06-19

**Authors:** Päivi Grönroos, Nur-E-Habiba, Kalle Salminen, Marja Nissinen, Tomi Tuomaala, Kim Miikki, Qiang Zhang, Nan Wei, Esko Kauppinen, Jarkko Eskola, Harri Härmä, Sakari Kulmala

**Affiliations:** 1 Department of Chemistry and Materials Science, School of Chemical Engineering, Aalto University, P.O. Box 16100, FI-00076 Aalto, Finland; nur.nur-e-habiba@aalto.fi (N.-E-H.); kalle.salminen@aalto.fi (K.S.); 2 Faculty of Medicine and Health Technology, Tampere University, FI-33720 Tampere, Finland; 3 Information Technology, Oulu University of Applied Sciences, FI-90250 Oulu, Finland; marja.nissinen@oamk.fi (M.N.); tomi.tuomaala@oamk.fi (T.T.); 4 School of Chemical Engineering, Aalto University, P.O. Box 16100, FI-00076 Aalto, Finland; kim.miikki@aalto.fi; 5 Department of Applied Physics, School of Science, Aalto University, P.O. Box 15100, FI-00076 Aalto, Finland; qiang.zhang@aalto.fi (Q.Z.); nan.wei@aalto.fi (N.W.); esko.kauppinen@aalto.fi (E.K.); 6 Labmaster Ltd, FI-20200 Turku, Finland; jarkko.eskola@labmaster.fi; 7 Department of Chemistry, University of Turku, FI-20014 Turku, Finland; harri.harma@utu.fi

**Keywords:** hot electron electrochemistry, electrochemiluminescence, ethyl cellulose, polystyrene, screen printed electrodes, bioaffinity assays, C-reactive protein, fluorescein isothiocyanate, terbium chelate electrochemiluminescence

## Abstract

Novel hot electron-emitting working electrodes and conventional counter electrodes were created by screen printing. Thus, low-cost disposable electrode chips for bioaffinity assays were produced to replace our older expensive electrode chips manufactured by manufacturing techniques of electronics from silicon or on glass chips. The present chips were created by printing as follows: (i) silver lines provided the electronic contacts, counter electrode and the bottom of the working electrode and counter electrode, (ii) the composite layer was printed on appropriate parts of the silver layer, and (iii) finally a hydrophobic ring was added to produce the electrochemical cell boundaries. The applicability of these electrode chips in bioaffinity assays was demonstrated by an immunoassay of human C-reactive protein (i) using Tb(III) chelate label displaying long-lived hot electron-induced electrochemiluminescence (HECL) and (ii) now for the first time fluorescein isothiocyanate (FITC) was utilized as an a low-cost organic label displaying a short-lived HECL in a real-world bioaffinity assay.

## 1. Introduction

Hot electron-induced electrochemiluminescence (HECL) is a method in which a variety of luminescent labels can be used in highly sensitive immunoassays on the basis of electrical excitation. HECL utilizes hot electrons normally produced by tunnel emission from thin insulating film-coated cathodes [1,2,3,4]. If the energy of the tunnel-emitted hot electrons is above the conduction band edge of water, electrons can enter the conduction band of water and are likely to become hydrated (e_aq_^−^) after thermalization and solvation [4,5,6,7,8]. When these energetic electrons react with dissolved oxygen and/or added co-reactants which are fast hydrated electron scavengers, secondary radicals are generated. These added co-reactants generate oxidizing radicals upon one-electron reduction (e.g., peroxodisulphate, peroxodiphosphate or hydrogen peroxide) or in the case of azide, upon one-electron oxidation. Thus, both extremely strong oxidants and reductants are produced, and normally one-electron redox reactions not obtainable occur and the excitation of several types of label compounds are enabled [4,9].

There are two major excitation routes: one usually being clearly dominant, and sometimes both excitation routes are almost equally significant, mostly depending on the lifetime and the redox properties of all the radical species involved in the system in aqueous solution. Label luminophores can first be one-electron reduced by a presolvated hot or hydrated electron to a corresponding radical species (Equation (1)) and then the formed luminophore anion radical is one-electron oxidized back to its original oxidation state by a strong one-electron oxidant leaving the luminophore in its excited state (Equation (2)) [10].

(1) $$L+e^{-}\to L^{•-}$$

(2) $$L^{•-}+Ox^{•}\to L^{*}+Ox^{-}$$

or alternatively, the luminophore is first one-electron oxidized to a radical species (Equation (3)) and then one-electron reduced by a hot presolvated electron or hydrated electron to its excited state (Equation (4)).

(3) $$L+Ox^{•}\to L^{•+}+Ox^{-}$$

(4) $$L^{•+}+e^{-}\to L^{*}$$

The excited luminophore finally emits light (Equation (5)) [10]:

(5) $$L^{*}\to L+hv$$

Various luminophores (e.g., Tb(III) chelates), organic fluorophores and Ru(bpy)_3_^2+^ have been studied when HECL excitation mechanisms and new electrode materials have been developed [11,12,13,14]. Tb(III) chelates have been the main label in bioaffinity assays of C-reactive protein [4,11,13,15], β_2_‑Microglobulin [16] and human thyroid stimulating hormone [12,17,18]. Some aromatic Tb(III) chelates exhibit high emission intensity and a relatively long luminescence lifetime which enables time-resolved detection and the use of materials generating relatively high background emission during cathodic excitation pulses in real-world assays, such as, oxide-covered aluminum electrodes. However, when the aim is to develop more inexpensive immunoassay methods, the use of low-cost luminophores with a short luminescence lifetime should be considered. Fluorescein isothiocyanate (FITC), is a photoluminescent label widely used in labeling different biomolecules due to the isothiocyanato group that couples with amino groups in protein and peptide chains [19,20].

Present composite electrodes consist of conductive carbon particles and polymer as insulating material on a printed silver layer. Insulating polymer material surrounds the carbon black particles and enables electrons to be transported through the thin composite layer with very low IR-loss, and finally hot electrons to be emitted at the electrode/solution interface. The main advantage of these novel electrodes over traditional insulating thin-film covered electrodes is their insensitivity to composite layer thickness and composition of the film [9,15,21,22]. Compared to thin insulating film-coated electrode materials, such as aluminum or silicon, composite electrodes are found to have a more stable and reproducible HECL output in a wider pH range, and most importantly, without considerable background emission [15,23,24]. Our research group has studied a few different composite electrode materials and found that the combination of (i) carbon black as a conductive material and (ii) polystyrene or ethyl cellulose as insulating binder materials is one of the best composite material to obtain a polymer layer doped with small conducting particles [15,24].

Polystyrene is one of the most commonly used supportive substrate material in a wide variety of bioanalytical protocols, such as enzyme-linked immunosorbent assays and fluoroimmunoassays [15]. We have also studied cellulose derivatives to explore more environmentally friendly composite cathode materials and found ethyl cellulose to be a very suitable insulating material for composite film-coated cathodes [24]. The low material cost of these composite electrodes combined with the possibility of manufacturing them in mass with low technology methods (e.g., screen or inkjet printing), makes them an ideal disposable electrode choice for things like point-of-care testing. In this study, we demonstrate the possibility to mass produce composite electrodes for electrochemiluminoimmunoassays (ECLIA) with an inexpensive screen printing method.

C-reactive protein (CRP) has been regarded as an early indicator of infectious or inflammatory conditions and as a universal biomarker for numerous diseases and disorders [25,26]. Thus, it was selected as a model analyte to demonstrate the feasibility of our novel electrodes and label materials to ECLIA. Fluorescein isothiocyanate was used as an example of organic electrochemiluminescent labels.

## 2. Materials and Methods

### 2.1. Preparation and Characterization of the Screen-Printed Electrodes

The composite solution mixture was first prepared. Polystyrene (PS, SKU 441147) and ethyl cellulose (EC, SKU 247499) were purchased from Sigma-Aldrich (St. Louis, MO, USA) and carbon black (CB, Vulcan XC72) was purchased from Cabot (Boston, MA, USA). Polystyrene and ethyl cellulose-carbon black solutions were made in benzyl alcohol (Acros Organics, Geel, Belgium) whose volatility properties are suitable for screen printing. The total mass concentration for PS-CB was 270 g/L, and the amount of carbon black was 40%, and for EC-CB it was 150 g/L and 30%, respectively. The mass rations of these solutions were chosen based on the needed viscosity for screen printing. Solutions were mixed with a Cole-Parmer ultrasonic homogenizer (amplitude 20%, 500 W, 20 kHz, Vernon Hills, IL, USA).

Two types of electrode geometries were studied and tested in printed cell chips: round-shaped electrodes (RS electrodes) and line/finger electrodes (LF electrodes) in which working and counter electrode lines were adjacent through the whole cell area like fingers of two hands.

**Figure 1:**
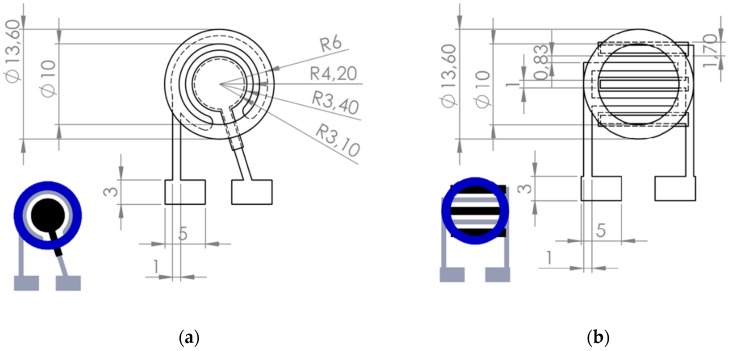
Schematic diagram of the screen-printed electrode chips (**a**) round-shaped (RS) and (**b**) line/finger (LF) electrodes. The anode was screen printed with silver (Ag) ink (grey). The cathode was screen printed with ethyl cellulose-carbon black (EC-CB) or polystyrene- carbon black (PS-CB) paste (black) on top of the Ag layer on working electrode areas. The hydrophobic ring was screen printed with dielectric paste (blue). Dimensions are given in mm. The cathode area (radius = 3.4 mm) of the RS is 46% of the sample area (r = 5 mm) and in LF electrode it is 40%.

The schematic pictures of the electrodes are presented in Figure 1. First, a layer of silver (Asahi silver ink, Asahi Chemical Research Laboratory CO., LTD Hachioji-city, Tokyo, Japan) was screen printed on the polymer substrate (Valox FR1 film, Saudi Basic Industries Corporation (SABIC), Riyadh, Saudi Arabia). Then the electrode sheet was annealed under 140 °C for 30 min. The first layer acted as a counter electrode and also provided electrical contact for the composite ink layer. The first composite layer was screen printed and the electrodes were cured in 120 °C for 30 min. The second composite layer was then screen printed on top of the first layer to ensure full coverage of the silver (Ag) layer, and then again cured in 120 °C for 30 min. The hydrophobic (HP) ring was then screen printed with dielectric paste (D2070423P5, Gwent Electronic Materials Ltd., Pontypool, Wales, UK) The net hole size was 60 µm and the thickness of wires was 45 µm (UX90-45; 230 wires/inch, NBC Meshtec Inc., Hino, Tokyo, Japan).

The EC-CB and PS-CB films were analyzed by scanning electron microscope (SEM, Zeiss Sigma VP, ZEISS International, Oberkochen, Baden-Württemberg, Germany) at 1 kV at Aalto University Nanomicroscopy Center (Aalto-NMC) premises, and by atomic force microscopy (AFM, Veeco Dimension 3100, Inc. Plainview, NY, USA) in tapping mode at Aalto NanoFab (Micronova).

Step height measurement of each layer on both EC and PS composite electrodes were analyzed using the Bruker Dektak XTL (Bruker Corporation, Billerica, MA, USA) stylus profiler at Micronova, Aalto University. Further topographical analysis of the surface was studied using an optical profiler (Filmetrics Profilm 3D, Micronova, Aalto University, Filmetrics Inc., San Diego, CA, USA).

### 2.2. HECL Measurements

Long-exposure-time photographs (1 s) of HECL were taken in a completely dark room with a Canon EOS 7D digital camera equipped with a Canon EF 100 mm f/2.8 L Macro IS USM lens. The camera and coulostatic pulse generator were turned on simultaneously.

The excitation pulses were generated with an in-laboratory-built coulostatic pulse generator [4]. The constant charge voltage pulses were −55 V with a charge of 31.5 µC for RS electrodes and −25 V with a charge of 67.2 µC for LF electrodes at a rate of 50 Hz. A sufficient amount of excitation cycles were recorded. The possibility of using direct current excitation was also shortly tested by using a simple DC (Direct Current) laboratory voltage supply. A photomultiplier tube module (PerkinElmer MH1993, 1364-H-064, Inc. Waltham, MA, USA) was used for optical detection with an interference filter, 550 nm for Tb(III) chelates and FITC, and the filter had a half-bandwidth of ca. 20 nm. A two-channel gated SR400 photon counter with a DC-300 MHz amplifier (both from Stanford Research System, Sunnyvale, CA, USA) was connected to the photomultiplier along with a Nucleus MCS-II multiscale card (Oxford Instruments Inc. Oak Ridge, TN, USA).

Tb(III) chelate, where the ligand was 4-(Isothiosyanatophenylethyl)(1-hydroxybenzene)-2,6-diyl)bis-(methylenenitrilo)tetrakis(acetic acid) (Tb(III)-1), was obtained from Turku University and fluorescein isothiocyanate (FITC) was purchased from Alfa Aesar (SKU LO9319, Ward Hill, MA, USA). In immunoassay a commercially available Tb(III) chelate and FITC was utilized as described below. All the HECL measurements were carried in 0.05 M Na_4_B_4_O_7_ buffer at pH 9.2 with 0.1 M Na_2_SO_4_ as the supporting electrolyte, and 0.01 M azide was chosen as the applied co-reactant to increase HECL intensity on the basis of our earlier studies [24,27,28].

### 2.3. Immunoassay of CRP

The working electrode was coated with primary anti-human CRP antibodies (anti-hCRP (anti-human C-reactive protein) clone 6405, Medix Biochemica) via physical adsorption by dispensing 50 μL of 5 μg mL^−1^ coating solution containing 50 mM Trizma base, 0.9% NaCl and 0.05% NaN_3_ (pH 7.7). The coating reaction was carried out overnight (>12 h) in room temperature. After the coating process, the electrodes were carefully dried with tissue paper. A total of 50 μL of saturation solution containing 6% D sorbitol and 0.1% bovine serum albumin in TSA (Tris Saline Azide) buffer (50 mM Tris, 0.05% NaN_3_, 0.9% NaCl, pH 7.7) was dispensed on the electrode surface. Following the saturation phase (3 h) the electrodes were dried and washed three times with 100 μL of 0.05 M Na_2_B_4_O_7_, 0.01 M NaN_3_, and 0.1 M Na_2_SO_4_ solution and the pH was adjusted to 7.9 with H_2_SO_4_. The heterogeneous immunoassays at screen printed electrodes coated with the primary antibodies was done by adding a mixture of human CRP (Scripps Laboratories Inc., San Diego, CA, USA) and FITC or Tb(III) chelate of 1 (p-isothiocyanatobenzyl)-diethylenetriamine N^1^,N^1^,N^2^,N^3^-pentaacetic acid (Tb(III)-2) (AD0029, PerkinElmer) labeled secondary antibodies (anti-hCRP clone 6404, Medix Biochemica Oy, Espoo, Finland) diluted in DELFIA (dissociation-enhanced lanthanide fluorescence immunoassay) assay buffer (PerkinElmer). The labeling of secondary antibodies was performed overnight at room temperature with a 70-fold excess of FITC or the Tb(III)-2 chelate in 0.5 M sodium carbonate at pH 9. The excess label was removed via gel filtration (NAP-10 column containing Sephadex G-25, GE Healthcare, Chicago, IL, USA) with a similar TSA buffer as above. Buffer exchanges were done with NAP-5 column containing Sephadex G-25 (GE Healthcare UK). Samples of hCRP were made by diluting the stock solution with TSA buffer containing 0.5% BSA (Bovine serum albumin), 3.5 mM CaCl_2_, 0.05% bovine γ-globulin and 0.01% Tween 20 (pH 7.7). A total of 15 μL of labeled antibody CRP solution was dispersed evenly over the electrode surface. The electrodes were then washed similarly as in the coating and saturating phases. The amount of FITC or Tb(III)-2 labeled secondary antibodies was 100 ng per electrode. All saturation and coating procedures were carried out in a closed humidor that had aqueous azide solution on the bottom to provide necessary vapor pressure.

## 3. Results

### 3.1. Characterization of the Electrodes

**Figure 2:**
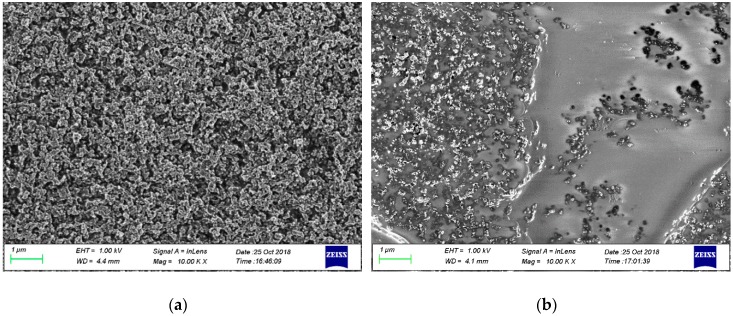
(**a**) EC-CB and (**b**) PS-CB electrode surfaces analyzed by SEM. Magnification of 10,000 times.

When the surface of EC-CB and PS-CB composites were analyzed by SEM (Figure 2), it was observed that the surface of EC-CB electrodes cathode was considerably more uniform than that of PS-CB electrodes. The same situation could already be seen when much less magnification was applied (Figure S1).

EC-CB and PS-CB electrodes were characterized also by AFM (Figure S2 EC-CB and Figure S3 PS-CB). Mean roughness of EC-CB was 54.6 nm (RMS (root mean square) roughness 67.0 nm) and of PS-CB was 58.0 nm (RMS roughness 74.2 nm).

**Figure 3:**
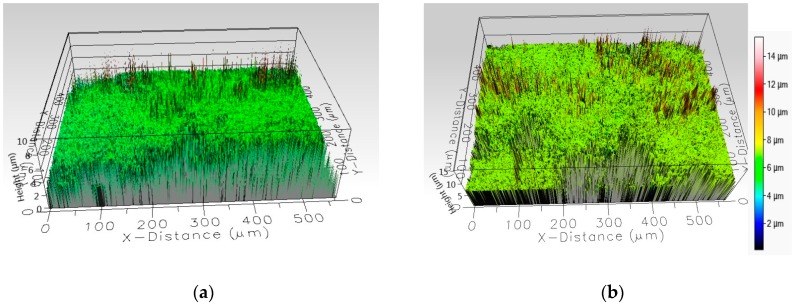
3D surface profile of (**a**) EC-CB and (**b**) PS-CB for topographical analysis, generated on Filmetrics Profilm 3D.

Step height measurement of each layer of RS EC-CB (Figure S4) and RS PS-CB electrodes and their surface topography were analyzed (Figure 3). The encompassed surface morphologies of the RS PS-CB electrodes layers are enclosed in the Supplementary Material (Figure S5).

Based on both 2D and 3D profiling, the average height of the CB layer on EC and PS electrodes was 6.78 (SD 1.30) µm and 7.7 (SD 0.68) µm, respectively. The calculated average thickness of the HP ring on EC and PS electrodes was 8.14 (SD 1.98) µm and 9.94 (SD 2.45) µm, respectively; whereas the bottom layers of Ag were 4.98 (SD 1.24) µm and 6.2 (SD 1.05) µm, respectively, on EC and PS electrodes.

### 3.2. Images of Luminescence during Cathodic Excitation

**Figure 4:**
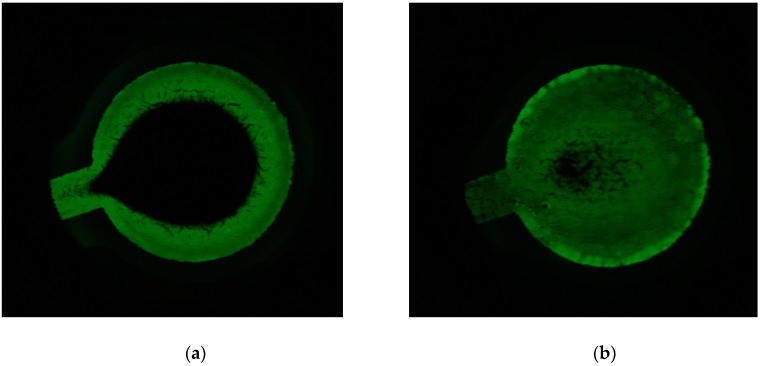
Long-exposure photographs, exposure time 1 s. −46 V, 31.5 µC, frequency 50 Hz. (**a**) 3 s and (**b**) 29 s after the beginning of the excitation with 0.01 NaN_3_ in 0.05 M Na_2_B_4_O_7_, 0.1 M Na_2_SO_4_. RS EC-CB electrode. The radius of the working electrode area was the same (3.4 mm) as in Figure 1.

The electrodes suitability to produce HECL was first tested with 10^−3^ M Tb(III)-1. Photographs of emission were taken to observe the spatial location of HECL emission on the working electrodes. In the beginning of the measurement luminophores located near the working electrode edge closer to the anode, were first excited. When pulse polarization (−45 V and 31.5 µC) was continued, the whole cathode area was eventually producing HECL with applied constant pulse parameters (Figure 4a). When voltage and charge were too low (e.g., −35 V and 9 µC), emission was seen only in the outer parts and it did not spread in the middle of the electrode during the measurement (Figure S6). With LF electrode geometry, a similar situation could not be observed, the whole area was in use already with lower voltage and charge (Figure S7). When the lights were on, the hydrogen production was observed in the same areas where the HECL intensity was highest (Figure S8).

Based on these observations, the pulse parameters were optimized (Figure S9) and the highest emission intensities were observed with RS EC-CB electrodes by applying −55 V and 31.5 µC.

### 3.3. Calibration Curves

**Figure 5:**
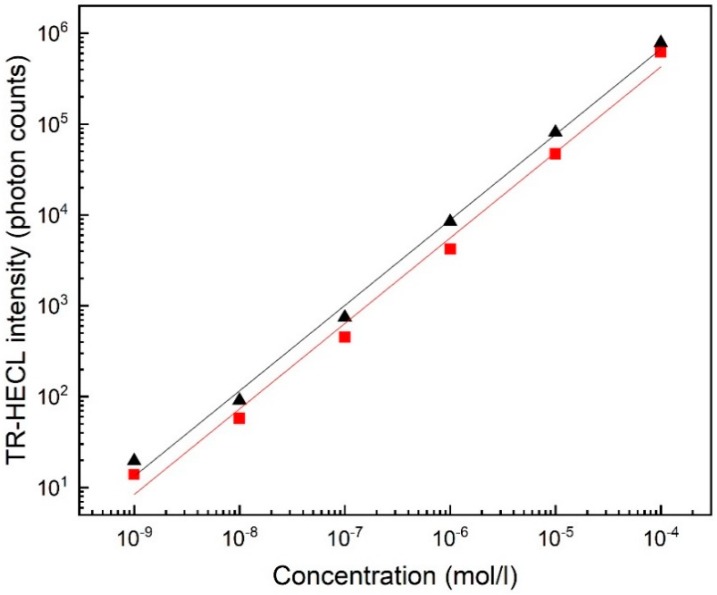
Calibration curve Tb(III)-1 with 0.01 NaN_3_ in 0.05 M Na_2_B_4_O_7_, 0.1 M Na_2_SO_4_. RS electrode. EC-CB (triangles), PS-CB (squares). Conditions: 2000 excitation pulses. Pulse charge 31.5 µC, voltage −55 V, frequency 50 Hz, delay 160 µs and gate 4 ms. Interference filter (550 ± 10 nm).

Blank-corrected calibration curves for Tb(III)-1 measured with EC-CB and PS-CB electrodes on RS chips are presented in Figure 5.

The detection limit (i.e., the blank subtracted calibration curve extrapolated to 3σ of blank emission) for Tb(III)-1 was 5 × 10^−9^ M with PS-CB and 1 × 10^−9^ M with EC-CB electrodes. Standard deviation (10^−6^ M Tb(III)-1) was 5% (*n* = 5) with PS-CB and 2% (*n* = 5) with EC-CB electrodes, which were also in the same level as spin-coated electrodes [15,24].

**Figure 6:**
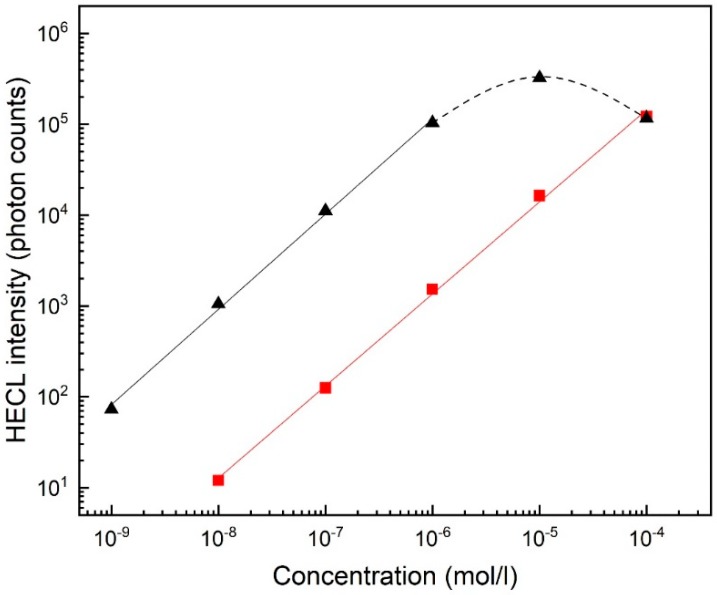
Calibration curves for fluorescein isothiocyanate (FITC) with 0.01 M NaN_3_ in 0.05 M Na_2_B_4_O_7_, 0.1 M Na_2_SO_4_. RS electrode. EC-CB (triangles), PS-CB (squares). Conditions: 500 excitation pulses. In case of EC-CB the light intensity was too high for photon counting above 1 micro molar solution. Pulse charge 31.5 µC, voltage −55 V, frequency 50 Hz, delay 0 s and gate 4 × 10^−5^ s. Interference filter (550 ± 10 nm).

Blank-corrected calibration curves for FITC measured with EC-CB and PS-CB electrodes are presented in Figure 6.

The detection limit (s/n = 3) of FITC was 8 × 10^−8^ M with PS-CB and 4 × 10^−10^ M with EC-CB electrodes. Standard deviation (10^−6^ M FITC) was 12% (*n* = 5) with PS-CB and 10% (*n* = 5) with EC-CB electrodes. The emission intensity of Tb(III)-1 and FITC as function ordinal numbers of excitation pulses is presented in the Supplementary Material (Figure S10). Emission of Tb(III)-1 was higher with PS-CB electrodes than with EC-CB electrodes in the beginning of the measurement; however, it started to decline after 250 pulses while EC-PS electrodes emission increased when 2000 pulses were measured and stayed stable for at least 2000 more pulses (Figure S10a). With FITC (Figure S10b), intensities with both electrode materials decreased from the beginning of the measurement, however, diminishing was slower in the case of EC-CB electrodes, and the intensity was all the time almost 100 times higher than with PS-CB (analogously to Figure 6).

Calibration curves of FITC were measured also with the LF electrodes with pulse voltage of −23 V and pulse charge of 67.2 µC (Figure S11) and the detection limit for FITC was observed to be in case of EC-CB 4 × 10^−9^ M and in case of PS-CB 8 × 10^−8^ M. These parameters were tested also for RS electrodes (Figure S12), and the detection limit for FITC was 2 × 10^−9^ M with EC-CB and 3 × 10^−8^ M with PS-CB electrodes.

The possibility of using direct current was tested with RS EC-CB electrodes (Figure S13). Linear calibration curve was spanning for the range of 10^−4^–10^−6^ M for FITC.

### 3.4. Immunonoassy of HumanCRP

The calibration curve of CRP was first measured with Tb(III)-2 as a label with RS EC-CB (Figure S14a) and RS PS-CB electrodes (Figure S14b). The detection limit (s/n = 3) of Tb(III)-2 label was 6 × 10^−1^ mg/L with PS-CB and 7 × 10^−2^ mg/L with EC-CB electrodes.

**Figure 7:**
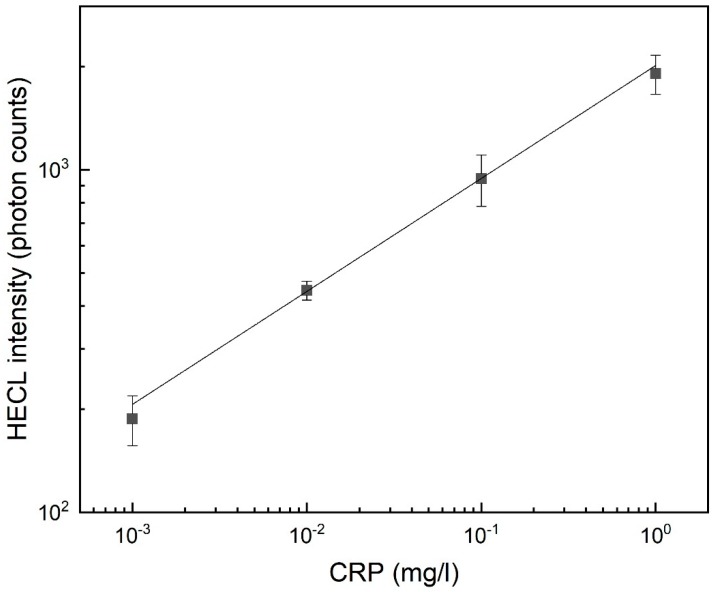
Blank subtracted calibration curves of C-reactive protein (CRP) with 15 min incubation time and 15 μL sample volume. 0.01 M NaN_3_ in 0.05 M Na_2_B_4_O_7_, 0.1 M Na_2_SO_4_. FITC 100 ng/cell. Conditions: 500 excitation pulses, pulse charge 31.5 µC, voltage −55 V, frequency 50 Hz, delay 0 µs and gate 4 × 10^−5^ s. Interference filter (550 ± 10 nm). Measured with RS EC-CB electrodes. Confidence bars calculated with 95% confidence level, *n* = 2.

The calibration curve of CRP was then measured with FITC as a label at RS EC-CB electrodes (Figure 7).

The calibration curve was linear over three orders of magnitude of concentration (0.001 to 1 mg/L) and the calculated detection limit (s/n = 3) was 6 × 10^−3^ mg/L. The calibration curve of CRP was also measured with LF electrodes with FITC (Figure S15) and it was linear over two orders of magnitude of concentration (0.01 to 1 mg/L) and the calculated detection limit was 5 × 10^−2^ mg/L.

## 4. Discussion

In HECL applications, the electrode chip and cell geometry and supporting electrolyte concentration dictate the usable and optimal cell voltage and current parameters. In immunoassays, the HECL method is surface area-sensitive, and also somewhat volume-sensitive in the detection step, since (i) the working electrode area determines how much immunocomplexes/other type of bio complexes can be collected on the working electrode surface, and (ii) the primary radicals can enter the solution only for about some tens of nm, or for about 200 nm at maximum [10].

When a thin layer cell is used to shorten the incubation time, and the HECL measurement is carried out in a thin layer cell, the present RS working electrode geometry is not usable since light would then be generated only at the outer edges of the working electrodes. In this kind of thin layer cell applications, the LF type of geometry with suitably high line numbers and line thicknesses (the line thickness of anode fingers can be smaller than that of cathode fingers) should be used when cathodes and anodes are integrated on the same plane. Or alternatively and more preferably, a thin layer cell is built (i) on top of the whole cathode area, (ii) separated by a printed spacer dictating the cell thickness (and in fact also the cell volume), and (iii) an anode such as indium tin oxide or graphene-coated plastic is used as an optically sufficiently transparent counter electrode.

In principle, nothing prevents the use of a conventional 3-electrode system in cases of electrodes integrated on the same surface plane in conventional potentiostatic control of the cell, but usually the compliance voltage of traditional potentiostats do not allow the use of traditional electrochemistry apparatus. Our coulostatic pulse generator gives a bit more reproducibility in the measurements but normal potentiostats used in two-electrode mode can be used almost equally well, if their compliance voltage is sufficiently high.

According to SEM studies, the PS-CB electrodes have some areas where the insulating PS and conductive CB are unevenly distributed (Figure 2). It may be possible that by using another solvent in printing, the applicability of PS-CB electrodes could be improved.

EC-CB (Figure S2) and PS-CB (Figure S3) electrodes were characterized also by AFM and the mean roughness of EC-CB (54.6 nm, RMS 67.0 nm) and of PS-CB (58.0 nm, RMS 74.2 nm) did not have significant differences so the actual surface areas of these materials can be assumed to be quite similar.

Based on both 2D and 3D profiling, the average height of the CB layer on EC and PS electrodes were very similar, ca. 7–8 µm. Thus, the printing conditions were rather good since we hoped to obtain about the same thicknesses for both composite materials. The calculated average thickness of the HP ring on EC and PS electrodes was also quite closely the same, 8–10 µm, so its reproducibility clearly was sufficient for the present purposes, when the hydrophobic ring was not used to determine the cell height of a thin layer cell. The bottom layers of Ag had a thickness of 5–6 µm which clearly produced the sufficient conductivity to the bottom layer.

With the presently studied printed cells for droplet incubation type of assays, the cell with a large RS working electrode needed a quite high pulse voltage (−55V, 100 µC for EC and −45 V, 80 µC for PS), before the whole cathode area was finally producing HECL with the applied constant pulse parameters (Figure 4). When the applied voltage and charge were having too low values, HECL emission was seen only in the outer edge parts of the working electrodes. For LF electrodes the whole area was producing HECL already with lower pulse voltage and pulse charge. The preferable measurement mode would probably be a pulse voltage scanning in the style of normal pulse polarography, but our instruments do not yet allow the use of this kind of detection mode. The use of RS working electrodes is preferable in droplet incubation assays since the whole cell area is utilized in a collection of bioaffinity complexes; on the other hand LF electrodes always waste the counter electrode area from the total cell area on the chip.

The detection limits for Tb(III)-1 using time-resolved detection was 5 × 10^−9^ M with PS-CB and 1 × 10^−9^ M with EC-CB electrodes and standard deviations of (10^−6^ M Tb(III)-1) 5% (*n* = 5) with PS-CB and 2% (*n* = 5) with EC-CB electrodes were almost as good as in our earlier studies. In case of earlier spin-coated electrodes, the detection limit of Tb(III)-1 was 2 × 10^−10^ M at cellulose acetate propionate EC-CB electrodes and 2 × 10^−10^ M at PS-CB electrodes [15,24]. Thus, extremely sensitive immunoassays do not yet seem obtainable with the present printed electrode chips since the detection limit of labels should be order of 1 × 10^−11^ M in cases of analytes such as the thyroid stimulating hormone (TSH).

FITC is a very inexpensive commercially available HECL-label whereas all the applicable Tb(III) chelates that are commercially available at the moment are very expensive. The detection limit (s/n = 3) of FITC with PS-CB (8 × 10^−8^ M) was considerably poorer than with EC-CB electrodes (4 × 10^−10^ M). This is possibly related to the difference in surface properties (hydrophobicity, etc.) of these two different polymers. Thus, FITC could be detected down to a lower concentration without time-discrimination than the presently used Tb(III) chelates using time-resolved detection, which was a bit surprising (Figure 5). However, reproducibility of both composite materials was in a similar level: standard deviation (10^−6^ M FITC) was 12% (*n* = 5) with PS-CB and 10% (*n* = 5) with EC-CB electrodes. Calibration curves of FITC were measured also with LF electrodes with smaller cathodic pulse voltage and smaller pulse charge than in case of RS electrodes, the detection limit of FITC being in case of EC-CB 4 × 10^−9^ M and in case of PS-CB 8 × 10^−8^ M. These parameters were applied also for RS electrodes (Figure S12). Detection limits were observed to be at EC-CB and PS-CB, 2 × 10^−9^ M and 3 × 10^−8^ M, respectively. This showed that while these were not optimal pulse parameters for RS electrodes the system still worked sufficiently well.

The possibility of using direct current was tested with EC-CB electrodes. The linear calibration curve spanned only in the range of 10^−4^—10^−6^ M. Thin oxide film-coated electrodes cannot be used at all under cathodic DC polarization due to the breakdown of the insulating film [29]. Thus, this is a good feature of the present composite electrodes. Measurement using DC polarization is instrumentally simple and it may be useful for some specific purpose later on (e.g., the present electrodes could be used to synthesize nanoparticles).

The calibration curve of hCRP was first measured with commercially available Tb(III)-2 as a label with both EC-CB and PS-CB electrodes. The detection limit (s/n = 3) was 6 × 10^−1^ mg/L with PS-CB electrodes (Figure S14a) and 7 × 10^−2^ mg/L with EC-CB electrodes (Figure S14b). PS-CB electrodes did not give as good results as could have been predicted on the basis of the measurements of Tb(III) chelates alone in the absence of proteins (Figure 5). With spin-coated PS-CB electrodes the calculated detection limit for CRP was 1.2 × 10^−3^ mg/L [15], which was much better than with the present case of screen-printed PS-CB electrodes.

The calibration curve of hCRP with FITC label with RS EC-CB electrodes was linear over three orders of magnitude of concentration (0.001 to 1 mg/L) and the calculated detection limit (s/n = 3) was 6 × 10^−3^ mg/L. In the case of RS PS-CB electrodes, the emission intensity was (analogously to Figure 6) only about 10% of the emission of that of EC-CB electrodes, so these could not be used equally well in this immunoassay. The calibration curve of hCRP with LF EC-CB electrodes using FITC as a label was linear over two orders of magnitude of concentration (0.01 to 1 mg/L) and calculated detection limit was 5 × 10^−2^ mg/L (Figure S15) which was similar to those obtained earlier with spin-coated EC-CB electrodes [24].

The anodic dissolution of silver from the counter electrode is not an issue in the time scale of the HECL experiment with the present printed cells, since the silver ions either do not have sufficient time to be diffused to working electrodes or they do not induce any adverse effects there. However, it would be reasonable to print the counter electrodes from materials that are not anodically dissolved.

The mass ratios of carbon black and polymer were chosen as a compromise between the highest emission intensity and the screen-printing properties of the composite material in a liquid state. The presently used solvent was selected due to its boiling point and printability properties of our composite ink. It seems that at least for the PS-CB electrodes, based on the SEM results, the printing conditions were not yet optimal. All in all, the present first attempt to obtain printed HECL cells for bioaffinity assays could produce fully usable disposable electrode ships that can be used in sensitive CRP assays.

On the basis of our present experiments FITC and probably many other organic luminophores [10] can be used as labels in practical immunoassays, when the low cost of assays is important. Then the time-resolved measurement system can be omitted, and hand-held instruments can be constructed to be very simple and less expensive. Thus, (i) pulsed excitation is just turned on and (ii) a certain fixed time of HECL is recorded and the intensities are plotted as a function of the concentration of the analyte.

However, if both short-lived HECL and long-lived HECL displaying labels (preferably emitting at a different wavelength range) are simultaneously applied, many types of possibilities for multi-parametric assays and referencing will emerge. Multianalyte determinations and internal standardization become possible within a single assay with only one incubation step per each of the printed cell wells on a multi-cell chip, if a light detector with at least some spectral and time-resolution is applied together with the pulsed excitation providing triggering to the light detection unit.

## 5. Conclusions

The presently studied printed RS and LF chips were found to be applicable in real-world immunoassays for an analyte important in point-of-care testing (POCT). Our RS chips are usable in bioaffinity assays where droplet incubation is used, and LF chips can preferably be used in thin layer cells. In thin layer cells, the counter electrode line width should be much smaller than that of the working electrode’s line width. In this way, a much higher working electrode area is obtained within the total area of the bottom of the cell. Thus, for disposable assay cartridges made of plastic, the LF chip types are the correct choices. Alternatively, one can use a simple separate composite film-coated cathode separated by a suitable spacer, which also dictates the cell volume, from the optically transparent counter electrode which allows the light detection through the counter electrode.

The performance of polystyrene as an insulating matrix in our screen-printing inks was poorer than could have been expected on the basis of our earlier spin-coating studies. For printed electrodes, it is not only sufficient that the polymer is having suitable insulating properties, but also (i) it must also have a good adhesion to the surface of the substrate used for printing and (ii) the composition of the amounts of conductive particles and polymer in a suitable solvent must be optimized also from the needs of the printing method, which surely was not yet obtained in this first effort of fabrication of printed electrode chips. Inkjet printing by material printers seems to be also usable and possibly a better method to print HECL electrode chips. Inkjet printing could be applied also to print the electrodes directly on the appropriate areas of the lower half of the disposable assay cartridge made of plastics, when the cartridge is composed of two parts that are finally combined together after coating of the electrodes with bio components (e.g., by gluing or laser welding). Cartridges are much more attractive from the point of the users than the droplet type incubation chips used in this study. However, screen-printed electrode chips can of course also be used as a part of an assay cartridge.

FITC label was usable, but Tb(III) chelates provide better analytical slope in the hCRP calibration curves. Thus, more expensive aromatic Tb(III) chelates should be used when the performance requirements are higher, and the low-cost FITC label is applicable when its performance is considered to be sufficient for the purpose. When the best aromatic Tb(III) chelates that are not yet commercially available are used, the detection limits even with the presently used printed electrode chips can be considerably lowered.

## Supplementary Materials

The following are available online at https://www.mdpi.com/1424-8220/19/12/2751/s1, Figure S1: Electrode surfaces analyzed by SEM, Figure S2: Surface roughness analyzed by AFM, Figure S3: Surface roughness analyzed by AFM, Figure S4: 2D surface profile of EC composite electrode, Figure S5: 3D analysis of surface morphology of RS PS-CB electrode, Figure S6: Long-exposure photographs of RS EC-CB electrodes with lower voltage and pulse charge, Figure S7: Long-exposure photographs of LF EC-CB electrodes with lower voltage and pulse charge, Figure S8: Long-exposure photographs of RS EC-CB electrodes with higher voltage and pulse charge, Figure S9: Tb(III)-1 HECL intensity as a function of voltage and charge. RS EC-CB and PS-CB electrodes, Figure S10: Tb(III)-1 HECL intensity as a function of ordinal number of excitation pulses, Figure S11: FITC calibration curves. LF EC-CB and PS-CB LF electrodes, Figure S12: FITC calibration curves. RS EC-CB and PS-CB LF electrodes, Figure S13: Calibration curve of FITC under direct current excitation. RS EC-CB electrodes, Figure S14: CRP calibration curve, Tb(III)-2-labeled secondary antibody. RS EC-CB and RS PS-CB electrodes, Figure S15: CRP calibration curve, FITC-labeled secondary antibody. LF EC-CB electrodes.
