# Supplementary material for: Immunoassays Based on Hot Electron-Induced Electrochemiluminescence at Disposable Cell Chips with Printed Electrodes
Source: Sensors (Basel). 2019 Jun 19;19(12):2751. doi: 10.3390/s19122751 (PMC6631709; doi:10.3390/s19122751)
Supplement: Supplementary file 1 [file sensors-19-02751-s001.pdf]

Supplementary material

Characterization of the electrodes and chips

The screen-printed composite electrodes were analyzed by SEM (Figure S1). PS-CB electrodes surface (right) had uneven areas while EC-CB (left) surface was quite uniform.

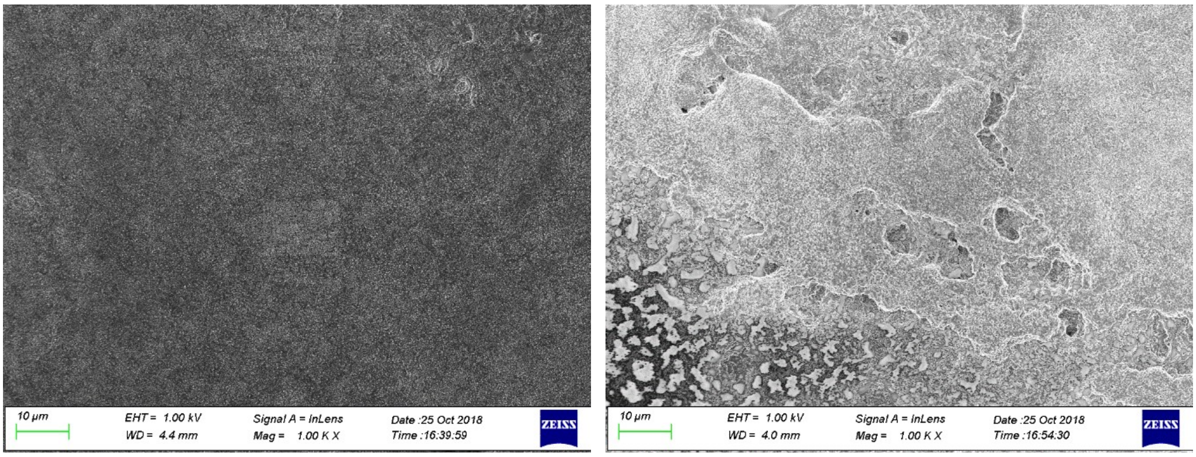

(a) (b)

Figure S1. (a) EC-CB and (b) PS-CB electrode surfaces analyzed by SEM. Magnification of 1000 times. RS electrode.

EC-CB (Figure S2) and PS-CB electrodes (Figure S3) were characterized also by AFM. Mean roughness of EC-CB was 54.6 nm (RMS roughness 67.0 nm) and of PS-CB was 58.0 nm (RMS roughness 74.2 nm) so there was not significant difference between the surface areas of these materials

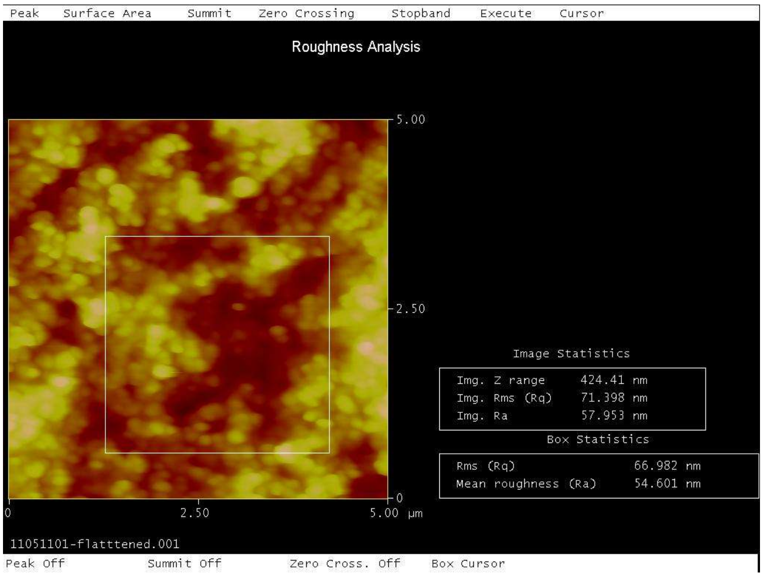

Figure S2. Surface roughness analysed by atomic force microscopy. RS EC-CB electrode.

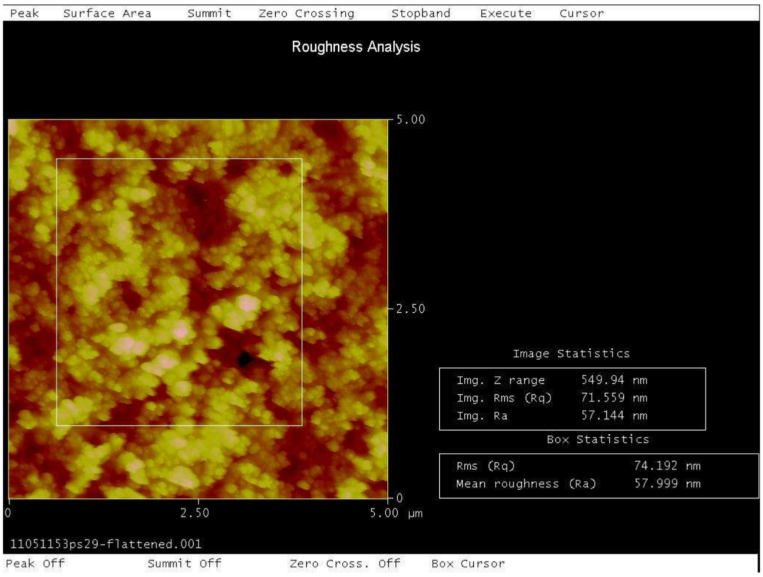

Figure S3. Surface roughness analysed by atomic force microscopy. RS PS-CB electrode.

The step heights of layers were measured by Stylus profilometer (Figure S4). Red (R) is reference area for the measurement, whereas green area (M) corresponds to the measured area of the layer.

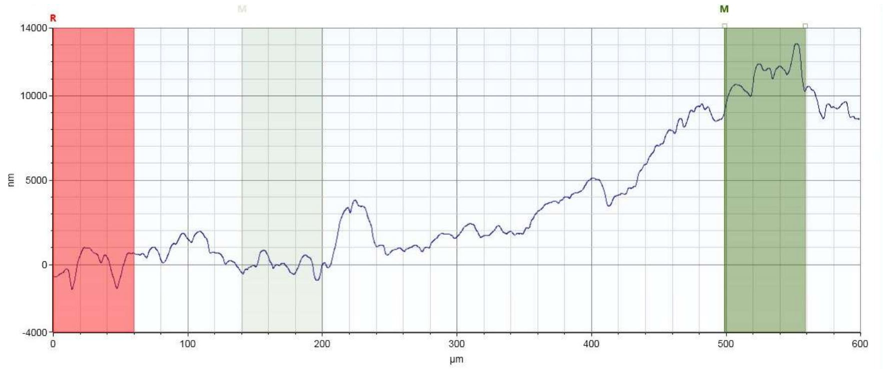

a.

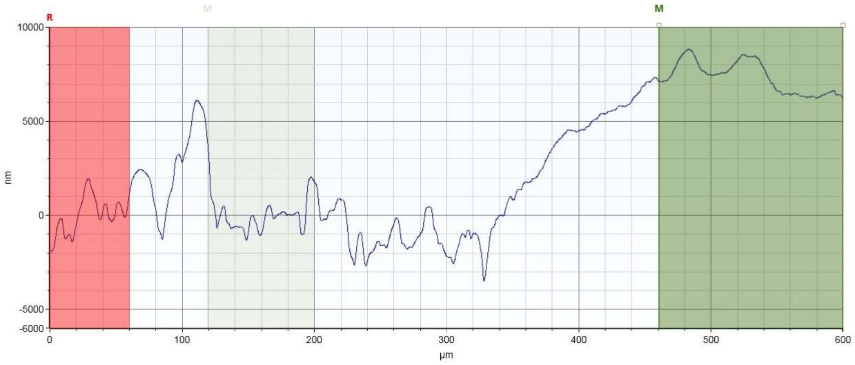

b.

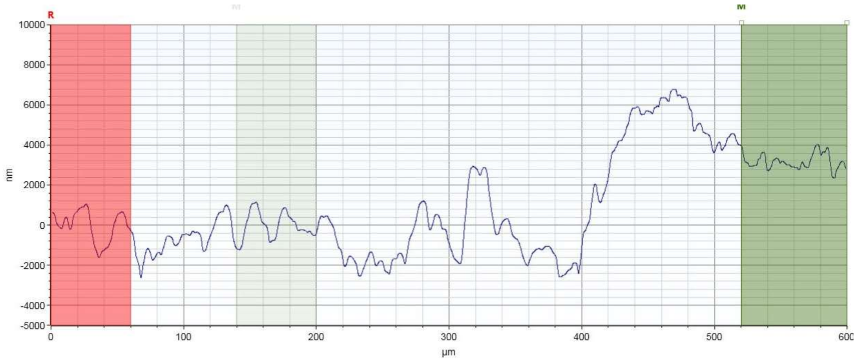

c.

Figure S4. 2D surface profile of EC composite electrode (a) CB circled working electrode, (b) HP ring, (c) Ag layer for step height measurement using stylus profiler.

The encompassed surface morphologies of the RS PS-CB electrodes layers are enclosed in the Figure S5.

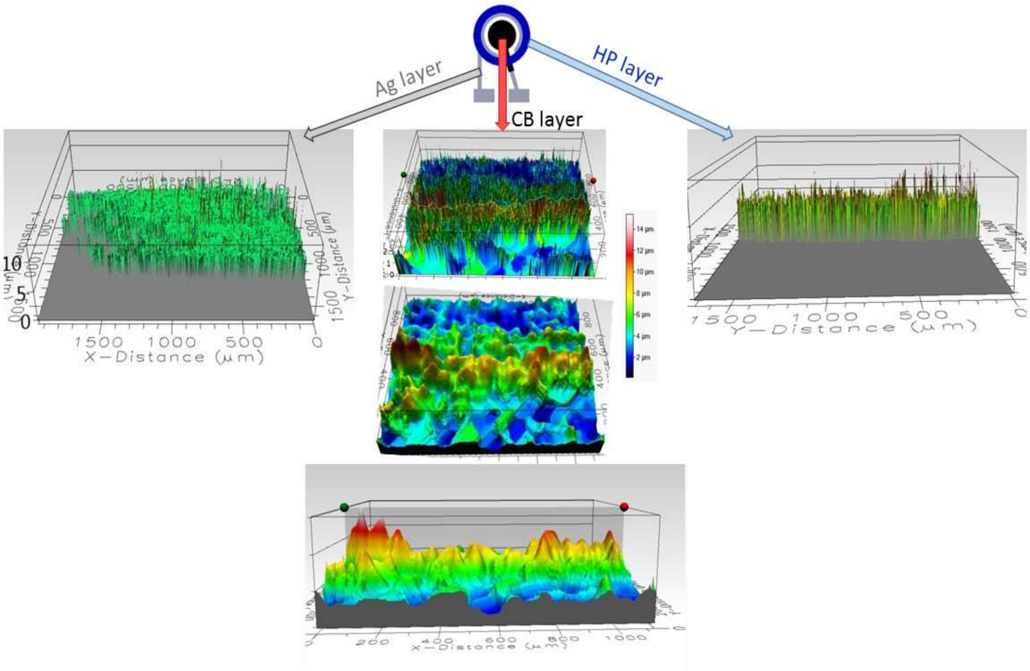

Figure S5. 3D analysis of surface morphology of RS PS-CB electrode using optical profiler.

Long exposure time photographs of HECL of Tb(III)-1 at EC-CB electrodes taken in completely dark room are presented in Figure S6 (RS electrode) and in Figure S7 (LF electrode).

Images of working electrodes during cathodic excitation

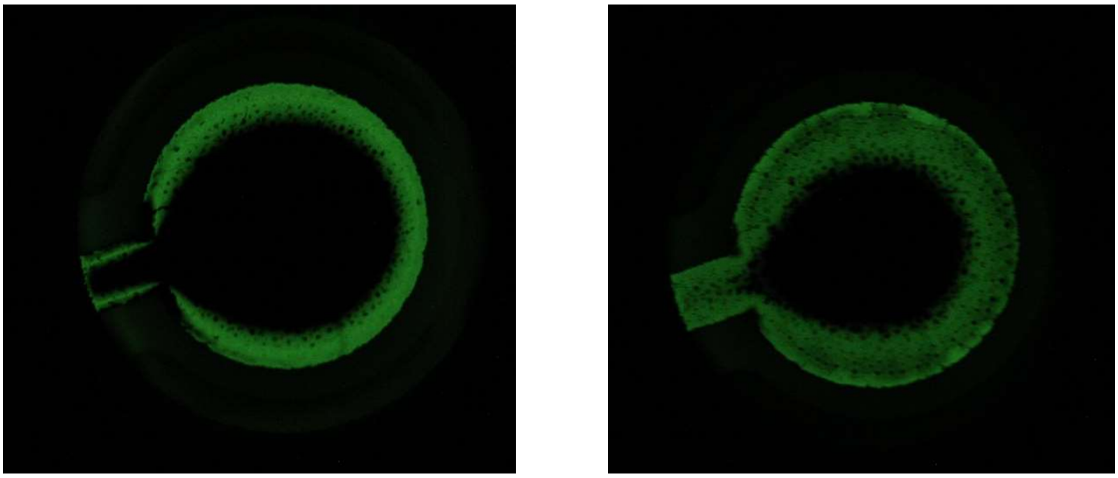

(**a**) (**b**)

Figure S6. Long exposure photographs, exposure time 1 s. Voltage – 35 V, pulse charge 9 μC, frequency 50 Hz. (a) 3 seconds and (b) 29 second after the beginning of the measurement. With 0.01 NaN_3 in 0.05 M $Na_2B_4O_7$, 0.1 M Na_2SO_4. RS EC-CB electrode.

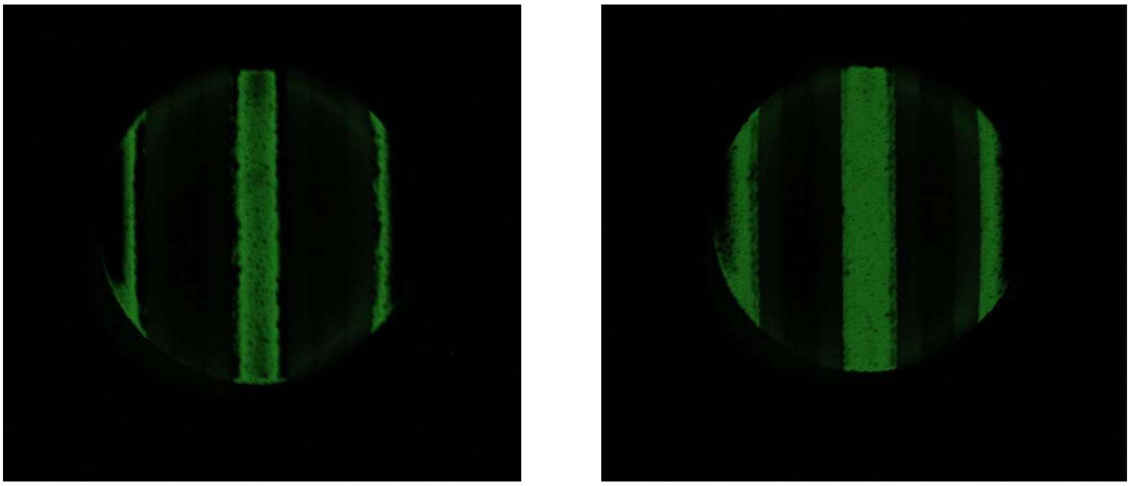

(**a**) (**b**)

Figure S7. Long exposure photographs, exposure time 1 s. Voltage – 35 V, pulse charge 9 μC, frequency 50 Hz. (a) 3 seconds and (b) 29 second after the beginning of the measurement. With 0.01 NaN_3 in 0.05 M $Na_2B_4O_7$, 0.1 M Na_2SO_4. LF EC-CB electrode.

Photos were taken when lights were on and the production of hydrogen can be seen in Figure S8.

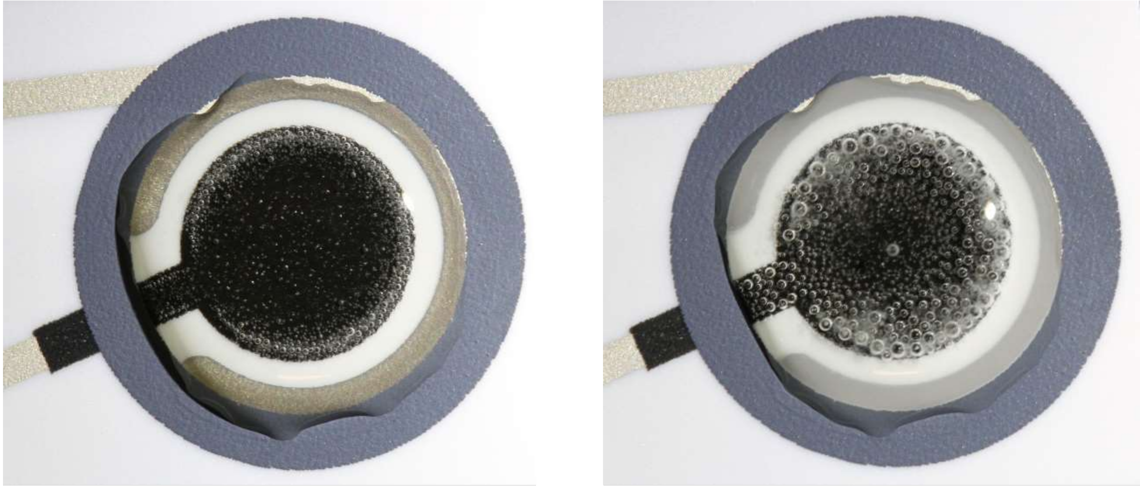

(**a**) (**b**)

Figure S8. Photographs of the electrode chips in room light. Voltage – 46 V, pulse charge - 31.5 μC, frequency 50 Hz. (a) 3 seconds and (b) 29 second after the beginning of the measurement. With 0.01 NaN_3 in 0.05 M $Na_2B_4O_7$, 0.1 M Na_2SO_4. RS EC-CB electrode.

Pulse parameters

The pulse parameters were studied for RS electrodes only (Figure S9).

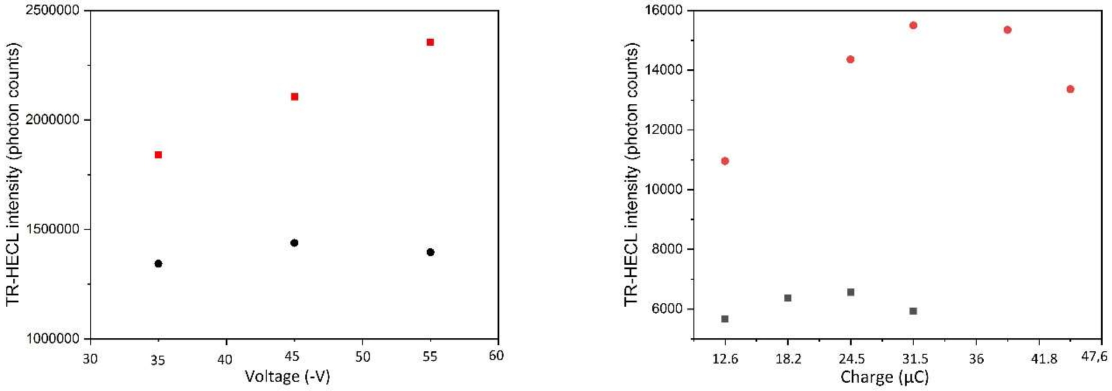

(**a**) (**b**)

Figure S9. (a) Intensity as a function of voltage. Pulse charge 31.5 μC (b) Intensity as a function of charge. Voltage - 55 V. RS EC-CB (red) and RS PS-CB electrodes (black). Tb(III)-1 10^{-4} M 0.01 NaN_3 in 0.05 M $Na_2B_4O_7$, 0.1 M Na_2SO_4. Conditions: frequency 50 Hz, (a) delay 160 μs and gate 4 ms. Interference filter (550±10 nm).

The emission intensity of Tb(III)-1 (a) and FITC (b) as a function ordinal number of excitation pulses are presented in Figure S10.

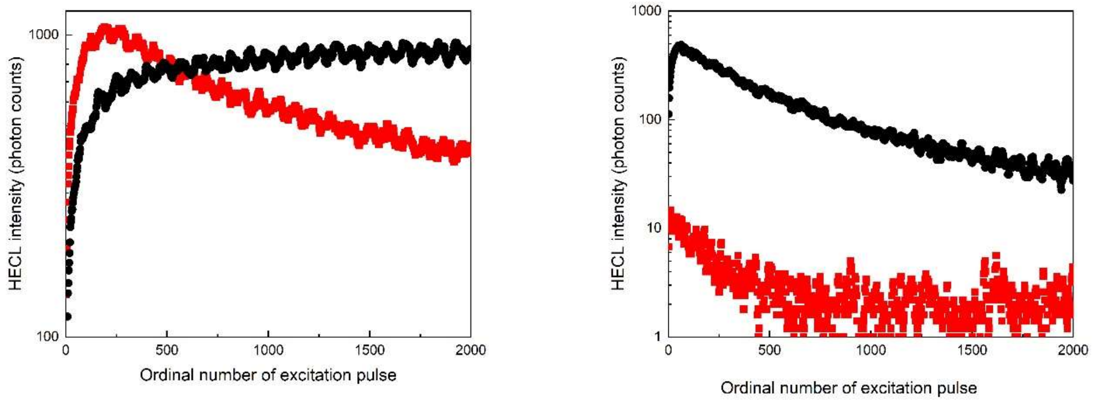

(**a**) (**b**)

Figure S10. (a) Tb(III)-1 10^{-4} M and (b) FITC 10^{-6} M with 0.01 NaN_3 in 0.05 M $Na_2B_4O_7$, 0.1 M Na_2SO_4. EC-CB (black), PS-CB (red). RS electrodes. Conditions: Pulse charge 31.5 µC , voltage - 55 V, frequency 50 Hz, (a) delay 160 µs and gate 4 ms. (b) delay 0 µs and gate $4 \cdot 10^{-5}$ ms. Interference filter (550±10 nm).

The emission intensity of Tb(III)-L with PS-CB electrodes (a, red) was higher in the beginning of the measurement and decreased quickly. Emission rised more slowly with EC-CB electrodes (a, black) but stayed more stable. When FITC was used as label, the intensity with PS-CB electrodes (Figure S10, b) rised and decreased more quickly than with EC-CB electrodes, and the intensity was less than 10 % of EC-CB electrodes intensity all the time.

Calibration curves

Calibration curves of FITC were measured also with the LF electrodes with - 23 V and 67.2 µC (Figure S11) and these parameters were tested also for RS electrodes (Figure S12).

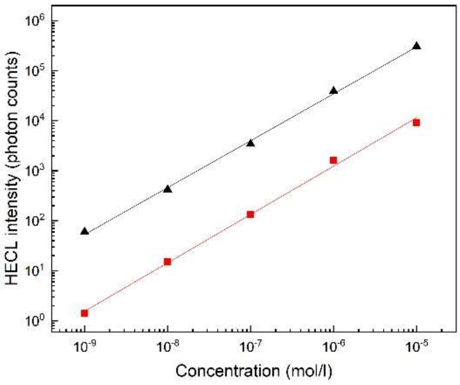

Figure S11. Calibration curves of FITC. EC-CB (triangles), PS-CB (squares) LF electrode. Pulse charge 67.2 µC, voltage - 23 V, frequency 50 Hz, delay 0 µs and gate $2 \cdot 10^{-4}$ s. Interference filter (550±10 nm).

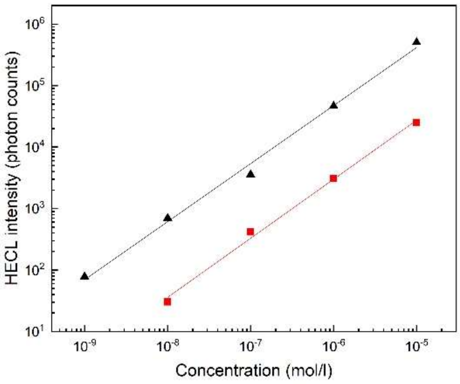

Figure S12. FITC. EC-CB (triangles), PS-CB (squares). RS electrode. Excitation: Pulse charge 67.2 μC, voltage - 23 V, frequency 50 Hz, delay 0 μs and gate $2 \cdot 10^{-4}$ s. Interference filter (550±10 nm).

The possibility of using direct current (DC) was tested with EC-CB electrodes (Figure S13). However, the calibration range was quite modest.

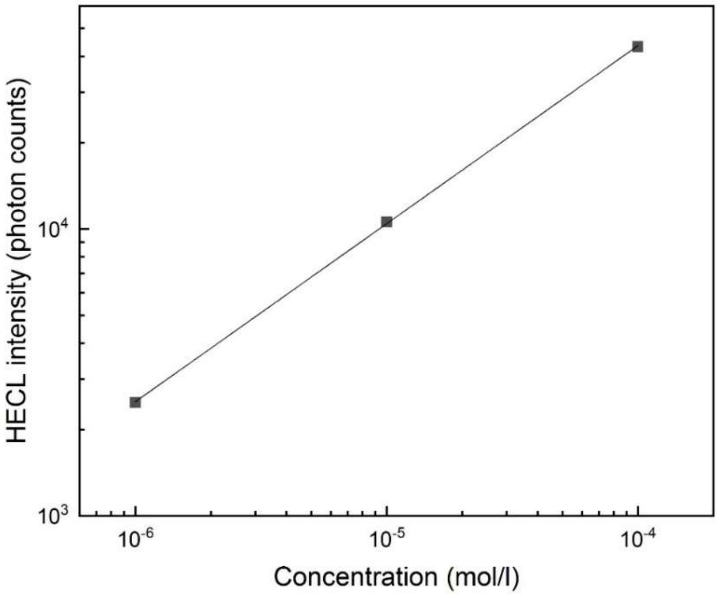

Figure S13. Calibration curve of FITC under direct current excitation. Conditions: - 20 V DC, measurement time 2.5 s, RS EC-CB electrodes. Interference filter (550±10 nm).

Immunoassay of CRP

Calibration curve of hCRP was measured with Tb(III)-2 as a label with RS EC-CB (Figure S14, a) and RS PS-CB electrodes (Figure S14, b). The detection limit (s/n=3) of Tb(III)-L was $6 \cdot 10^{-1}$ mg/l with PS-CB and $7 \cdot 10^{-2}$ mg/l with EC-CB electrodes.

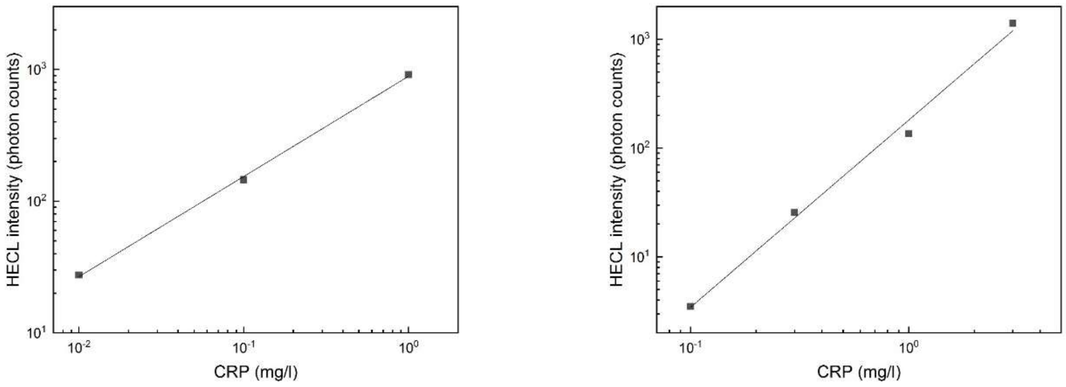

(**a**) (**b**)

Figure S14. hCRP calibration curve, Tb(III)-2-labelled secondary antibody (a) RS EC-CB electrode (b) RS PS-CB electrode. Conditions: TR HECL was recorded for (a) 2000 and (b) 500 excitation pulses, Pulse charge 31.5 μC, voltage - 55 V, frequency 50 Hz, delay 160 μs and gate 4 ms. Interference filter (550±10 nm).

Calibration curve of CRP was measured with FITC as a label with LF EC-CB electrodes (Figure S15). The detection limit (s/n=3) was $5{\cdot}10^{-2}$ M.

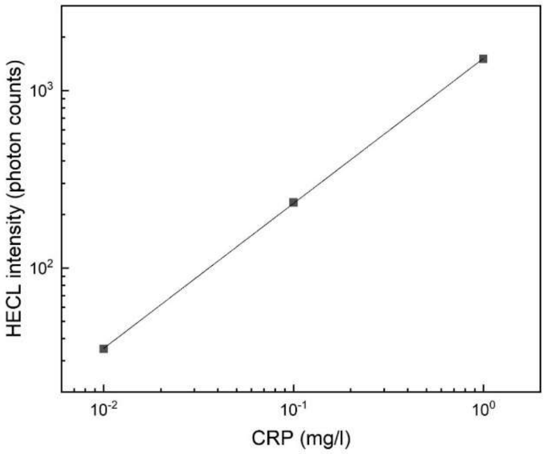

Figure S15. hCRP calibration curve in LF EC-CB electrodes, FITC-labelled secondary antibody, excitation: Pulse charge 67.2 μC, voltage – 23 V, frequency 50 Hz, delay 0 μs and gate $2{\cdot}10^{-4}$ s Interference filter (550±10 nm).
